# Silencing speckle-type POZ protein by promoter hypermethylation decreases cell apoptosis through upregulating Hedgehog signaling pathway in colorectal cancer

**DOI:** 10.1038/cddis.2016.435

**Published:** 2016-12-29

**Authors:** Xiaofei Zhi, Jinqiu Tao, Lei Zhang, Ran Tao, Lilin Ma, Jun Qin

**Affiliations:** 1 Department of General Surgery, The Affiliated Hospital of Nantong University, Nantong 226001, China; 2 Department of General Surgery, Affiliated Drum Tower Hospital of Nanjing University Medical School, Nanjing 210029, China; 3 Department of General Surgery, The First Affiliated Hospital of Nanjing Medical University, Nanjing 210029, China

## Abstract

Epigenetic silencing of tumor suppressors contributes to the development and progression of colorectal cancer (CRC). We recently found that speckle-type POZ protein (SPOP) was significantly downregulated and the inactivation of SPOP promoted metastasis in CRC. This study aimed to clarify its epigenetic alteration, molecular mechanisms and clinical significance in CRC. Our results revealed that the core region of *SPOP* promoter was hypermethylated in CRC tissues and its methylation was correlated with poor survival. Transcription factor RXRA had a vital role in the regulation of *SPOP* gene. The data indicated that DNA methylation at −167 bp of the *SPOP* gene altered the binding affinity between transcription factor RXRA and *SPOP* promoter. Moreover, SPOP was found to associate with Gli2 and promoted its ubiquitination and degradation in CRC. Consequently, the expression level of Hh/Gli2 pathway-related apoptotic protein Bcl-2 was decreased and the function of resisting cell death was inhibited in CRC. It suggests that methylation status of *SPOP* promoter can be used as a novel epigenetic biomarker and a therapeutic target in CRC.

Colorectal cancer (CRC) is the third most common cancer and the fourth most common cancer cause of death globally.^1^ DNA hypermethylation has now been linked to specific steps in the adenoma–carcinoma sequence, and is found to have a vital role in the initiation and progression of CRC.^2^ The assessment of methylated genes in colorectal cancers has also revealed a unique molecular subgroup of colorectal cancers called CpG Island Methylator Phenotype (CIMP) cancers.^3, 4, 5^ Global DNA hypermethylation is simultaneously accompanied by transcriptional silencing of tumor suppressor or DNA repair genes.^6^ For instance, six genes (*CDH1*, *CDKN2A/p16*, *ESR1*, *HLTF*, *ITGA4* and *p14*) are methylated during progression of aberrant crypt focus to adenoma, and four genes (*CXCL12*, *ID4*, *IRF8* and *TIMP3*) are frequently methylated in the late stages of the adenoma–carcinoma sequence.^7, 8, 9, 10^

Recently, speckle-type POZ protein (SPOP), an E3 ubiquitin ligase adaptor, is found to be frequently repressed in prostate cancer and gastric cancer, implying that SPOP acts as a tumor suppressor.^11, 12^ Our previous study revealed that SPOP was significantly downregulated and the repression of SPOP promoted metastasis in colorectal cancer.^13^ Notably, systematic whole-genome sequencing demonstrates that the *SPOP* gene is mutated in 6–15% of human prostate cancer, and functional analyses show that these are all loss-of-function mutations.^14, 15^ However, there are only about 2% of colorectal cancers harboring SPOP mutation, which means the aberrant low expression level should account for the repression of SPOP function in colorectal cancer, instead of somatic mutation.^16^ However, how this tumor suppressor gene *SPOP* is downregulated in colorectal cancer remains to be unknown.

Ubiquitin-dependent proteolysis has an important role in the regulation of a variety of cellular processes, including cell proliferation, differentiation and apoptosis.^17^ Ubiquitin (Ub) is attached to target proteins by a cascade enzyme system consisting of Ub-activating enzyme (E1), conjugating enzyme (E2) and ligating enzyme (E3). In this process, E3 enzyme determines the substrate specificity. Among the various E3 enzymes, Cul3-based ligase is known to regulate the cell apoptosis.^18^ SPOP has been identified to function as a substrate-specific adaptor that binds to Cul3 (ref. 19) and exert tumor-promoting or tumor-inhibiting effects depending on the specific substrate in different tumors.^20, 21^ Recently, SPOP has been proven to be responsible for full-length Gli2 ubiquitination and proteolysis.^20^ Hedgehog (Hh) signaling pathway is crucial in tissue-patterning during embryonic development and tumorigenesis.^22^ Binding of Hh ligands to Patched (PTCH) relieves Smoothened (SMO), and subsequently initiates the cascade signaling to activate the transcriptional factors (Gli1, Gli2 and Gli3).^23^ However, the function and downstream substrate of SPOP remains unknown in colorectal cancer.

In this study, we found that hypermethylation of *SPOP* promoter induces the transcriptional repression and decreased cell apoptosis in colorectal cancer. We suggested that demethylation of *SPOP* promoter region can be used as the novel epigenetic therapy for colorectal cancer.

## Results

### The promoter region of the *SPOP* gene is hypermethylated in CRC

**Figure 1:**
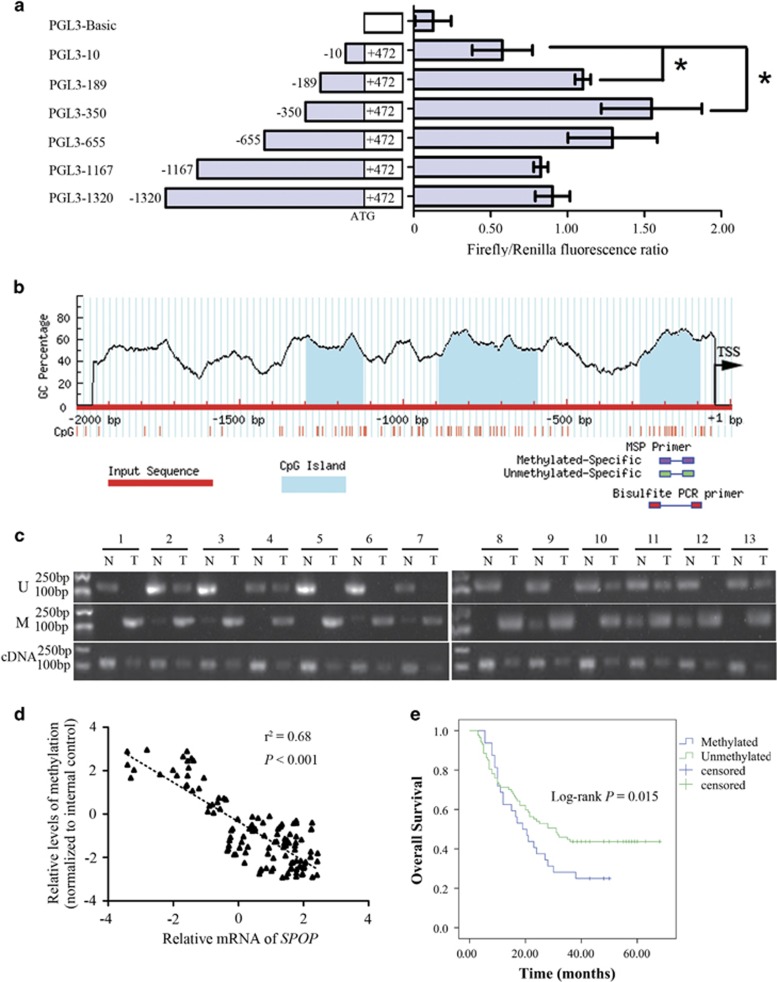
Hypermethylation of the core part in *SPOP* promoter in CRC. (**a**) Luciferase assays of five *SPOP* promoter region constructs were carried out. The ratio of Renilla luciferase to Firefly luciferase was calculated for each experiment. The mean value for each test construct was normalized to the activity of the empty vector. (**b**) Scheme for the location of the CpG islands in the transcription start region in *SPOP* gene. The CpG sites are indicated by vertical red lines. The regions for MS-PCR and BSP are indicated. TSS, translation start site. (**c**) Representative results of methylation analysis by MS-PCR and expression analysis by quantitative PCR in CRC tissues (T) and adjacent normal tissues (N). U, unmethylation; M, methylation. (**d**) correlation analysis of the MSP results. (**e**) The survival analysis of CRC patients with methylated and unmethylated *SPOP* promoter. *P<0.05. Data represent the results from three independent experiments

To evaluate the impact of DNA methylation pattern on transcriptional regulation of *SPOP* gene, we first analyzed which specific region within the *SPOP* gene promoter is critical to the regulation of gene transcription. Serial deletion constructs of the *SPOP* gene promoter region were generated. As shown in Figure 1a, the region from −350 to +472 had the strongest promoter activity, and the region from −350 to −10 seemed to be the core part accounting for increasing promoter activity (*P*<0.001).

Next, we scanned the *SPOP* promoter for potential regions of DNA methylation, and a distinct CpG island was observed in the promoter region between −276 and −96 bp, which was within the core part of the promoter (Figure 1b). Methylation-specific PCR (MSP) of this region in CRC and adjacent tissues showed *SPOP* promoter methylation was found in 31 out of 118 CRC patients, and was associated with TNM stage and lymph node metastasis (Figure 1c,Table 1). Quantitative PCR revealed a negative correlation between the extent of *SPOP* promoter methylation and gene expression (Figure 1c). Moreover, there was a strong negative correlation between the extent of *SPOP* promoter methylation and gene expression (Figure 1d), indicating SPOP expression was silenced by promoter region hypermethylation in CRC.

The prognostic value of the *SPOP* promoter methylation was detected with Kaplan–Meier survival analysis. As shown in Figure 1e, the overall survival was longer in the *SPOP* unmethylation group compared to the methylation group (*P*=0.015), suggesting that *SPOP* promoter methylation may serve as a poor prognostic predictor in CRC.

### Hypermethylation of the specific CpG sites within *SPOP* promoter decreases transcriptional activities

**Figure 2:**
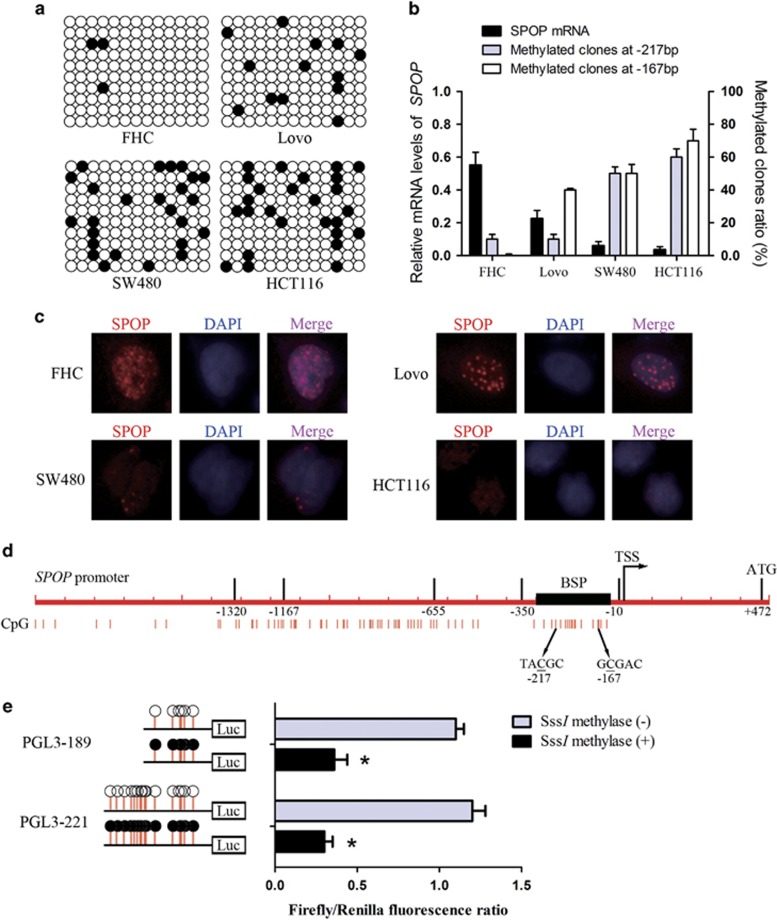
Correlation between hypermethylation of the specific CpG sites within *SPOP* promoter and transcriptional activities. (**a**) Bisulfite sequencing analysis of the methylation status of the *SPOP* promoter in the indicated cell lines. Open and closed circles indicate the unmethylated and methylated CpG dinucleotides, respectively. BSP region was from from −271 to −27 bp and included 13 CpG sites. (**b**) The mRNA levels of *SPOP* and methylated clones ratio at −217 and −167 CpG sites. (**c**) Immunofluorescence assays were used to examine the SPOP protein distribution in these cells. (**d**) Scheme for generating two deletion fragments of the *SPOP* promoter region and the CpG sites at −167 and −217 bp. (**e**) Treatment of two luciferase constructs with *SssI* methylase *in vitro*. *P<0.05. Data represent the results from three independent experiments

To provide a detailed DNA methylation pattern within the core part of *SPOP* promoter, we performed bisulfite sequencing (BSP) in three CRC cell lines Lovo, SW480, HCT116 and one human colon normal epithelium cell line FHC. The sequencing region was from −271 to −27 bp and included 13 CpG sites. As shown in Figure 2a, the analyzed promoter region was significantly hypermethylated in CRC cell lines compared with colon normal epithelium cell. In particular, CpG sites at −217 and −167 bp showed the highest methylation level. Moreover, methylation ratio of these two sites was negatively correlated with SPOP mRNA expression (Figure 2b), suggesting that the expression of SPOP might be downregulated by DNA methylation of the two CpG sites. Furthermore, we performed immunofluorescence assays to examine whether differential level of methylated SPOP is correlated with cytoplasmic *versus* nuclear SPOP protein distribution in these cells. As shown in Figure 2c, SPOP protein was predominantly located in the nucleus of FHC cells and Lovo cells, as well as in the cytoplasm with a small amount, whereas SPOP protein was markedly downregulated and was only located in the nucleus of SW480 cells and HCT116 cells, which was negatively correlated with methylation levels of SPOP.

To further identify which CpG site is responsible for the methylation-associated inactivation of the *SPOP* gene, two constructs of the *SPOP* gene promoter region were treated with *SssI* methylase *in vitro*, and then transfected into FHC cells (Figures 2d and 2e). Increased methylation of promoter constructs induced significant repression of promoter activity in comparison with untreated constructs. Specifically, the excessive region of PGL3-221 compared with PGL3-189 failed to affect promoter activities with or without *SssI* methylase treatment. These data indicate that an increased level of DNA methylation at −167 bp of the *SPOP* gene is a key factor in the regulation of *SPOP* gene transcription.

### Transcription factor RXRA binding affinity is altered by a DNA methylation pattern in the *SPOP* promoter

**Figure 3:**
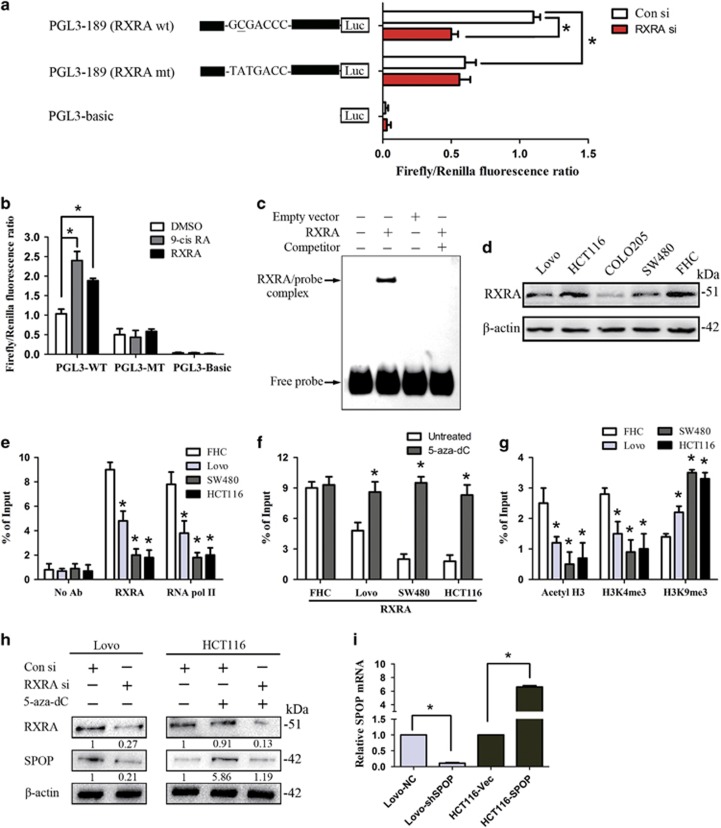
Influence of −167 CpG site methylation on the binding affinity between transcription factor RXRA and *SPOP* promoter. (**a**) Promoter analysis using the TRANSFAC database indicated that the sequence around −167 CpG site is the consensus sequence of transcription factor RXRA (5′-GCGACCC-3′). Luciferase assays of this region construct were carried out. wt, wild type; mt, mutant type. Con si, Control siRNA; RXRA si, RXRA siRNA. (**b**) Luciferase assays were performed to examined the response of wild-type and mutant reporters to 9-cis RA and exogenous RXRA overexpression. (**c**) EMSA assay was performed to confirm the interaction between RXRA and *SPOP* promoter. RXRA protein was obtained when pcDNA3.1(−)A-RXRA or empty vector as DNA template was used in the TNT T7/SP6 Coupled Reticulocyte Lysate System. Competitor probes were added at 100-fold molar excess. (**d**) Western blot was used to examine the RXRA protein level in cell lines. The numbers are referring to the predicted protein sizes. (**e**) Chromatin immunoprecipitation quantitative reverse transcription polymerase chain reaction was used to assay for RNA polymerase II and RXRA. (**f**) As assayed by chromatin immunoprecipitation with RXRA antibody, enrichment of RXRA in *SPOP* promoter was rescued with 5-aza-dC treatment. (**g**) Enrichment of active histone modification markers acetyl-histone H3 (acetyl-H3) and trimethyl histone H3 (Lys4) (H3K4 me3), and inactive marker trimethyl histone H3 (Lys9) (H3K9 me3) were detected. (**h**) The protein levels of RXRA and SPOP were detected with western blotting. The numbers are referring to the predicted protein sizes. (**i**) Real-time PCR was used to study the transfection efficiency. *P<0.05. Data represent the results from three independent experiments

Promoter analysis using the TRANSFAC database indicated that the sequence around −167 site in the *SPOP* gene promoter is the consensus sequence of transcription factor RXRA (5′-GCGACCC-3′, −168 to −162 bp). To confirm the interaction between RXRA and the *SPOP* gene, the luciferase assay was carried out. As shown in Figure 3a, *SPOP* promoter activity was significantly reduced upon knockdown of RXRA and mutation of RXRA binding element. Besides, we also examined the response of wild-type and mutant reporters to 9-cis RA and exogenous RXRA overexpression. As shown in Figure 3b, FHC cells were pretreated with DMSO, 9-cis RA (100 nM, 16 h) and RXRA vector (48 h). The luciferase activity of the wild-type RXRA binding element was significantly enhanced in 9-cis RA and RXRA vector group compared with the control group. Moreover, the increase of luciferase activity was abolished in the reporter containing mutant-type RXRA binding element. These results indicated that the sequence around −167 site in the *SPOP* gene promoter is binding element for transcription factor RXRA. To strengthen this conclusion, an oligonucleotide probe and an *in vitro*-translated full-length RXRA protein were used in EMSA assays. Full-length RXRA protein exhibited high-affinity and sequence-specific binding to the proposed binding element (Figure 3c).

Before performing the chromatin immunoprecipitation-qRT-PCR (ChIP-qPCR) assay, we examined the RXRA protein levels in cell lines (Figure 3d). ChIP-qPCR assay for RXRA binding showed that the enrichment of RNA polymerase II and RXRA on the *SPOP* promoter sequence in CRC cell lines was significantly higher than that in colon normal epithelium cell line FHC (Figure 3e). In addition, the enrichment of RXRA was increased in CRC cell lines by treatment with 5-aza-dC (Figure 3f). Intriguingly, the enrichment of active histone modification marks such as acetyl-histone H3 (acetyl-H3) and trimethyl histone H3 (Lys4) (H3K4 me3) in CRC cell lines were lower than that in FHC cells, while the inactive trimethyl histone H3 (Lys9) (H3K9 me3) mark was higher in CRC cell lines were lower than that in FHC cells (Figure 3g). Transfection of RXRA–targeted short interfering RNA (siRNA) in Lovo cells induced a reduction in the level of SPOP expression, while treatment with 5-aza-dC in HCT116 cells increased the expression of SPOP (Figure 3h).

### SPOP promotes cell apoptosis and anoikis *in vitro*

**Figure 4:**
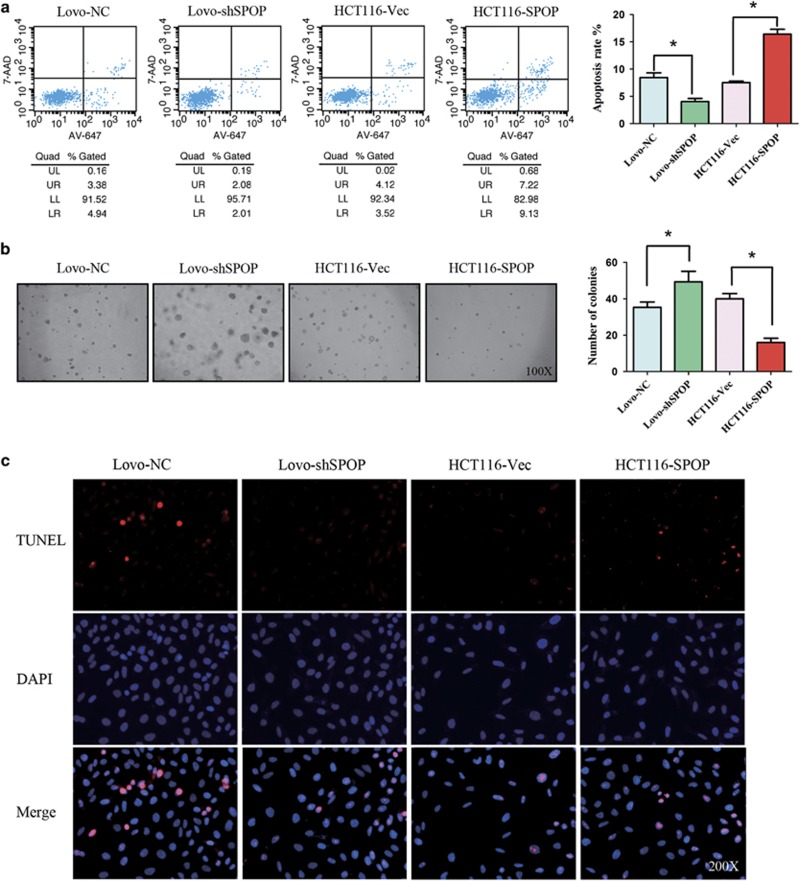
Acceleration effect of SPOP on cell apoptosis and anoikis *in vitro*. (**a**) Cell apoptosis was measured with flow cytometry. The cells were stained with Alexa Fluor 647 Annexin V and 7-AAD. Upper (late apoptotic cells) and lower (early apoptotic cells) right quadrants are counted. (**b**) Cell anoikis was measured with soft agar assay. (**c**) Cell apoptosis was measured with Terminal-deoxynucleoitidyl Transferase Mediated Nick End Labeling (TUNEL). *P<0.05. Data represent the results from three independent experiments

Our previous study has reported that SPOP acts as a tumor suppressor by inhibiting cell proliferation and migration in CRC. To further investigate how epigenetic silencing of the *SPOP* gene affects CRC progression due to resisting cell death, knockdown or overexpression of the *SPOP* gene was induced in Lovo and HCT116 cells (Figure 3i). When the *SPOP* gene was downregulated in Lovo cells, flow cytometry results showed that cell apoptosis was significantly decreased (Figure 4a) and soft agar assay revealed that cell anoikis was also decreased (Figure 4b). TUNEL assay results confirmed that knockdown of SPOP marked decreased cell apoptosis (Figure 4c). In contrast, overexpression of *SPOP* in HCT116 cells significantly increased cell apoptosis and anoikis (Figure 4).

### SPOP inhibits tumor growth and promotes tumor death *in vivo*

**Figure 5:**
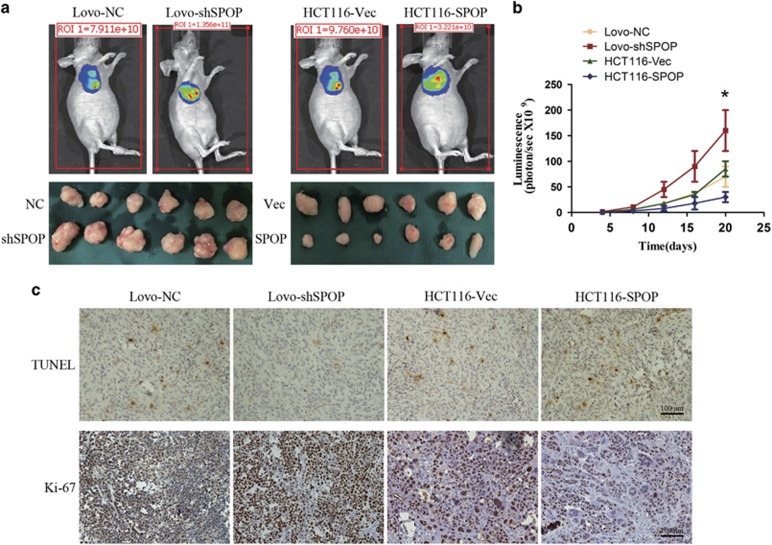
Effect of SPOP on tumor growth and tumor death *in vivo*. (**a**) Representative bioluminescence imaging and xenografts. (**b**) Tumor growth curves were indicated by bioluminescence (photons/s). (**c**) The apoptosis level and Ki-67 expression level of subcutaneous tumors were detected with TUNEL and IHC, respectively. The representative images are from the same tumor samples. *P<0.05. Data represent the results from three independent experiments

To further study the effects of SPOP on CRC progression, *in vivo* experiments were performed via the subcutaneous transplantation of CRC cells into BALB/c nude mice. Bioluminescence imaging results showed that SPOP significantly inhibited tumor growth (Figures 5a and b). Moreover, Ki-67 staining of tumor xenografts confirmed that SPOP markedly decreased the fraction of Ki-67-positive tumor cells (Figure 5c). The apoptosis levels of tumor xenografts were detected using TUNEL. The data showed that the ability of resisting cell death in Lovo-shSPOP cells was enhanced compared with control cells, while overexpression of SPOP in HCT116 promoted tumor cell apoptosis (Figure 5c).

### SPOP mediates the ubiquitination and degradation of Gli2 and modulates apoptosis signaling

**Figure 6:**
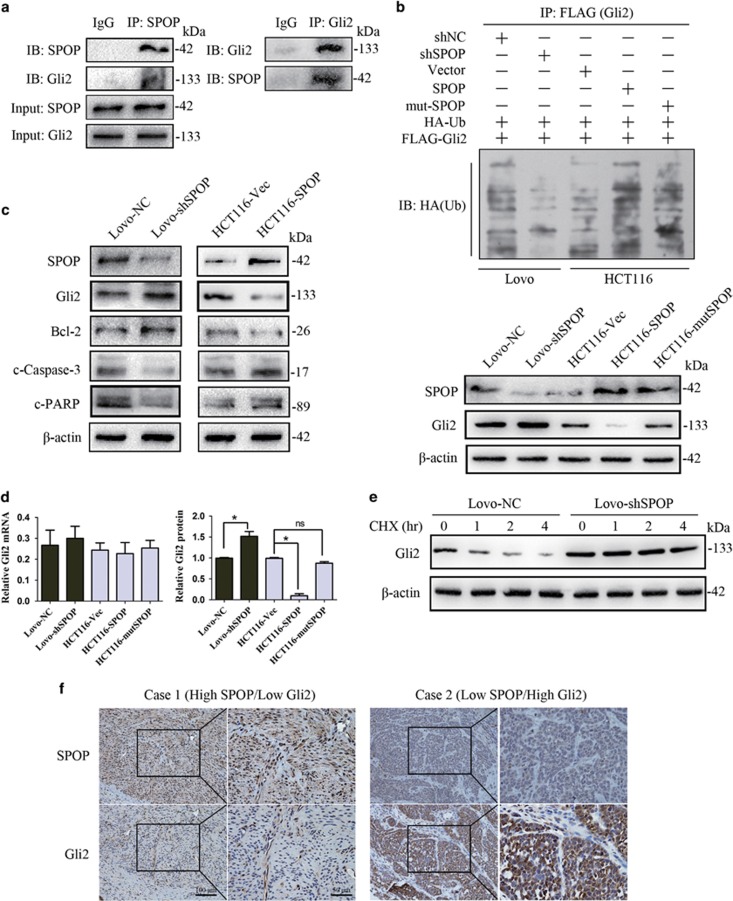
SPOP mediating the ubiquitination and degradation of Gli2 and modulating apoptosis signaling. (**a**) The interaction between SPOP and Gli2 was detected by co-immunoprecipitation. Goat anti-rabbit IgG is used as negative control. (**b**) Ubiquitination assays of Gli2 were performed by co-transfection of FLAG-Gli2 and HA-Ub. After immunoprecipitation by FLAG antibodies, HA antibody was used to detect poly-ubiquitinated Gli2. (**c**) The expression levels of Hh/Gli2 pathway-related apoptotic proteins Bcl-2, cleaved Caspase-3 and cleaved PARP were detected with western blotting. (**d**) The mRNA levels of Gil2 were detected by qRT-PCR and the protein levels were quantified based on (**b**) lower panel. (**e**) Gli2 stability was detected by cycloheximide protein degradation rate experiments. (**f**) The representative photos of SPOP and Gli2 expression in CRC tissue microarray. The number of CRC patients is 118. The correlation between SPOP and Gli2 expression was examined by Spearman correlation test. The numbers are referring to the predicted protein sizes. The experiment was repeated three times. *P<0.05

To explore the underlying mechanism that SPOP suppresses the ability of resisting cell death in CRC, we investigated the relationship of SPOP and Hh/Gli2 pathway, which is generally accepted to be involved in anti-apoptosis. Co-immunoprecipitation assay showed that SPOP interacted with Gli2 in Lovo cells (Figure 6a). Ubiquitination Assay revealed that ubiquitinated Gli2 was decreased after downregulation of SPOP in Lovo cells (Figure 6b, upper panels). Meanwhile, Gli2 expression was effectively rescued (Figure 6b, lower panels). In contrast, when overexpression of SPOP in HCT116 cells, ubiquitinated Gli2 was increased and Gli2 expression was reduced (Figure 6b, upper panels). Although Gli2 protein levels in SPOP mutant cells were marked increased compared with SPOP-overexpression cells (Figure 6b, lower panels), mutation in the MATH domain of SPOP lightly attenuated its degradation activity on Gli2 protein (Figure 6b, upper panels). Taken together, the data suggested that the mechanism of SPOP-mediated Gli2 degradation was due to enhanced ubiquitination of Gli2.

To further investigate the downstream signaling of SPOP/Gli2 interaction, we detected the expression levels of Hh/Gli2 pathway-related apoptotic proteins Bcl-2, cleaved Caspase-3 and cleaved PARP. As shown in Figure 6c, repressed SPOP expression in Lovo cells resulted in the increased expression of Bcl-2 and decreased expression of cleaved Caspase-3 and cleaved PARP, whereas enhanced SPOP expression in HCT116 cells lead to the opposite results.

To confirm the ubiquitination regulation of Gli2 mediated by SPOP, we investigated the transcription level and protein level of Gli2 (Figure 6d). The mRNA levels of Gil2 were detected by qRT-PCR and the protein levels were quantified based on Figure 6b lower panel. Our results revealed no significant changes in the levels of Gli2 mRNA. Conversely, the protein level of Gli2 was significantly altered in SPOP-knockdown or SPOP-overexpression cells. In addition, the stability of Gli2 protein was detected by cycloheximide protein degradation rate experiments. As shown in Figure 6e, cycloheximide treatment of cells for up to 4 h resulted in a remarkable reduction in the steady-state levels of the Gli2 protein, whereas knockdown of SPOP caused an elevation in Gli2 protein abundance and there was no change in protein levels compared with controls. These results indicated that SPOP suppressed Gli2 protein expression through post-transcriptional repression. Last, we also detected the expression pattern of SPOP and Gli2 in tissue microarray with 118 CRC patients. As shown in Figure 6f, the results showed a statistically significant correlation between SPOP and Gli2 levels (Spearman correlation test, *r*=−0.364, *P*<0.001).

## Discussion

Hypermethylation is a common event in the silencing of tumor suppressor genes in most cancer types.^24^ Although CRC pathogenesis is also associated with epigenetic alterations such as DNA hypermethylation and dysregulation of micro RNAs,^1^ epigenetic biomarkers for the diagnosis or prognosis of CRC have not yet been established. In our previous study, we suggested the potential role of *SPOP* in progression of CRC.^13^ However, the underlying mechanism remains unknown. In this study, we revealed that the tumor suppressor gene *SPOP* was inactivated by DNA methylation of its promoter region. We also examined the relationship between hypermethylation of the *SPOP* promoter and the clinical phenotypes of CRC patients. Intriguingly, the methylated *SPOP* promoter was associated with poor overall survival, suggesting that it may be a valuable prognostic biomarker in CRC. In particular, bisulfite sequencing is a simple and useful technique for single-base methylation pattern with a highly integrated resolution. The data indicate that DNA methylation at −167 bp of the *SPOP* gene is a key factor in the regulation of *SPOP* gene transcription by altering transcription factor RXRA binding affinity. To our knowledge, this is the first report to identify a critical regulatory region within the *SPOP* promoter.

As reported in several previous studies, SPOP is a novel substrate-specific adaptor binding to Cul3 and determines the specificity of ubiquitin-dependent proteolysis. However, as SPOP has a wide spectrum of substrates, it exerts tumor-promoting or tumor-inhibiting effects depending on the specific substrate in different tumors.^20, 21^ Our results suggested a novel function of SPOP, that is, its apoptosis-promoting effect in CRC. The function of resisting cell death is important for the progression of CRC. Intriguingly, SPOP was found to associate with Gli2 and promoted its ubiquitination and degradation in CRC. Knockdown of SPOP increased the expression of Hh/Gli2 pathway-related anti-apoptotic proteins Bcl-2. Moreover, the immunohistochemistry assay result indicated a negative correlation between SPOP and Gli2 levels in CRC tissue microarray, which supports a hypothesis that the ubiquitination regulation of Gli2 is mediated by SPOP.

DNA methylation may affect gene transcription by two major mechanisms. First, the methylated DNA and methyl-CpG-binding domain (MBD) proteins can recruit histone deacetylases and other chromatin remodeling proteins to the locus, which leads to the change of the chromatin structure. Second, methylated cytosine residue can interfere with the binding between transcription factors and their binding elements in the promoter region.^25^ We demonstrated that RXRA is a vital transcriptional regulator of the *SPOP* gene and the methylation of CpG site within the RXRA binding element interfered with the binding affinity. Treatment with the DNA demethylating agent 5-aza-dC rescued the binding affinity. Moreover, our results suggested that H3K9 me3, which is a transcriptional repression mark was enhanced, while the enrichment of active histone marks acetyl-H3 and H3K4 me3 was reduced. These interactions contributed to gene silencing caused by DNA methylation.

In some other types of cancer with a high frequency of SPOP mutation, such as prostate and endometrial cancers with 8–14% rate of SPOP mutation, little is known about how SPOP mutations influence the stability of its substrates. The 374-residue SPOP protein contains three domains: an N-terminal MATH domain (residue 28–166) that recruits specific substrates, an internal BTB domain (residue 190–297) that binds Cul3 and a C-terminal nuclear localization domain (residue 365–374). To date, most identified mutation sites were located in the MATH domain, which determines the specific substrates, such as Ser14Leu in exon 2, Tyr87Cys in exon 4 and Phel133Leu in exon 5. Thus, these mutations in the MATH domain of SPOP might have influence on its substrate degradation. Indeed, Theurillat *et al.*^15^ analyzed changes in the ubiquitin landscape induced by SPOP mutants (Tyr87Cys or Phel133Leu) in prostate cancer cells and revealed that DEK and TRIM24 were consistently upregulated by SPOP mutants. Here, we generated a common SPOP mutant (Tyr87Cys) by site mutation. Although Gli2 protein levels in SPOP mutant cells were markedly increased compared with SPOP-overexpression cells, mutation in the MATH domain of SPOP lightly attenuated its degradation activity on Gli2 protein. Further studies are needed to investigate the underlying mechanisms.

Easy-to-detect, robust and automatable tumor markers are heavily requested for early detection of CRC. To date, the established noninvasive stool tests, guaiac fecal occult blood tests (gFOBTs) and fecal immunochemical tests (FITs) are considered effective.^26^ However, the hypothetical best detection method for CRC is still missing. Promoter hypermethylation of tumor-related genes can be used as a sensitive marker for CRC early diagnosis, prognosis prediction and therapeutic target.^27, 28^ Thus, recent developments for CRC screening have focused on the epigenetic basis of cancer development. Our results showed that hypermethylation of *SPOP* promoter was detected in 31/118 CRC patients and this methylation-associated inactivation of the *SPOP* gene contributed to the CRC cell anti-apoptosis, suggesting that hypermethylation of *SPOP* could be a potential marker for CRC diagnosis. Further studies are needed to clarify the possible screening methods combining hemoglobin test and SPOP methylation test for CRC.

In conclusion, our study shows that hypermethylation of a CpG island in the promoter region of the *SPOP* gene regulates its transcriptional level by affecting the binding affinity between transcription factor RXRA and its binding element in CRC. In addition, SPOP regulates the ubiquitination and degradation of Gli2 in CRC, and consequently decreased the expression of Hh/Gli2 pathway-related apoptotic proteins. These data imply that the differential methylation pattern of the *SPOP* promoter in CRC and non-CRC tissues is a potential target for a novel epigenetic therapeutic reagent.

## Materials and methods

### Patients and specimens

Colorectal cancer tissue was obtained from 118 patients with colorectal cancer who underwent radical resection in the Affiliated Hospital of Nantong Medical University from 2008 to 2010. All the patients were diagnosed pathologically according to the criteria of the American Joint Committee on Cancer. Clinicopathological details are provided in Table 1. The study protocols were approved by the Ethical Committee of the Affiliated Hospital of Nantong University. All animal work was approved by the Ethical Committee of Affiliated Hospital of Nantong University. Written informed consents were obtained before specimen collection.

### Tissue microarray construction and immunohistochemistry

Colorectal cancer tissue microarray (TMA) was constructed by the National Engineering Centre for Biochip, Shanghai, China. The number of CRC patients is 118. A standard protocol was used for immunostaining of the TMAs. Immunoreactivity was scored independently by two pathologists using a semi-quantitative immunoreactivity score (IRS).^29^ The IRS was calculated by combining the quantity score with the intensity score. The quantity score documented the percentage of immunoreactive cells as 1 (0–25%), 2 (26–50%), 3 (51–75%) and 4 (76–100%). The intensity score documented the intensity of immunostaining as 0 (negative), 1 (weak), 2 (moderate) and 3 (strong). Multiplication of quantity score and intensity score resulted in an IRS ranging from 0 to 12. Under these conditions, samples with IRS 0–4 and IRS 6–12 were classified as low and high expression, respectively.

### Bisulfite modification, methylation-specific PCR (MSP) and bisulfite sequencing (BSP)

Genomic DNA was isolated from human colorectal cancer tissue and cells. The EZ DNA Methylation-Gold kit (Zymo Research, Orange, CA, USA) was used for bisulfite treatment according to the manufacturer's instructions. Primer pairs that specifically amplified either methylated or unmethylated sequences spanning the CpG island were used. SPOP-MSP-M-F: 3′-TTTTTGAGTAGTTGGGATTATAGGC-5′, SPOP-MSP-M-R: 3′-ACACTTTAAAAAACCGAAACGAA-5′, SPOP-MSP-U-F: 3′-TTTTGAGTAGTTGGGATTATAGGTGT-5′, SPOP-MSP-U-R: 3′-TCAACACTTTAAAAAACCAAAACAA-5′. Normal blood genomic DNA untreated and treated with *SssI* methylase (New England Biolabs, Beverly, MA, USA) was used as fully methylated and fully unmethylated controls in all PCRs, respectively.

For BSP, bisulfite-treated DNA was amplified by PCR using the primers: SPOP-BSP-F: 5′-TGTTGTGAAATGTTTTTAATTGT-3′, SPOP-BSP-R: 5′-CCTAAATTTCCCTACTCCTCCTC-3′. PCR products were purified with a Wizard SV Gel and PCR Clean-up System (Promega, Madison, WI, USA). Then PCR products were cloned into a pGEM-T easy vector (Promega). Ten colonies were chosen randomly for plasmid DNA extraction using a Promega Spin Mini kit (Promega) and were then sequenced by an ABI 3130 Genetic Analyzer (Applied Biosystems, Foster City, CA, USA).

### Real-time PCR

Total RNA was extracted from the cells using Trizol plus kit (Takara, Kusatsu, Japan). First-strand cDNA synthesis was performed using Promega kit. Synthesized cDNA was used for qRT-PCR analysis using SYBR Premix Ex Taq Kit (Takara) based on the manufacturer's instructions. *β*-actin was used as an internal control. All the procedures were performed in triplicate.

### Western blotting

Total protein was isolated from cells using cell extraction buffer (50 mM Tris–HCl (pH 7.4), 150 mM NaCl, 1% Triton X-100, 0.1% SDS, 1 mM EDTA and protease inhibitor cocktail). Protein concentrations were measured using a BCA Protein Assay kit (Pierce, Rockford, IL, USA). Antibodies against SPOP (Abcam, Cambridge, UK), Gli2 (Abcam, Cambridge, UK), RXRA (Abcam, Cambridge, UK), *β*-actin (Abcam, Cambridge, UK), Bcl-2 (Abcam, Cambridge, UK), cleaved Caspase-3 (Abcam, Cambridge, UK), cleaved PARP (Abcam, Cambridge, UK) were used. All immunoblots were performed with triplicate.

### Construction of recombinant plasmids and lentivirus production

The full-length ORF of *SPOP* (1125 bp, NM_001007226.1) was amplified from cDNA of FHC cells. The primers were as follows: F: 5′-AGAGAATTCATGTCAAGGGAAATCTTTGC-3′, R: 5′-AGAGGATCCTTAGGATTGCTTCAGGCGTT-3′. To construct the lentivirus production containing SPOP, the ORF of SPOP was subcloned into the pLenti-CMV-GFP vector (Addgene, Cambridge, MA, USA).

The synthesized DNA fragments encoding the short-hairpin RNA (shRNA) used for the knockdown of endogenous SPOP were inserted into the pGPU6/GFP/Neo vector (GenePharma, Shanghai, China). The sequences of the shRNAs were as follows: shSPOP, 5′-AACGCCTGAAGCAATCCTACTCGAGTAGGATTGCTTCAGGCGTT-3′, NC, 5′-TTCTCCGAACGTGTCACGTCTCGAGACGTGACACGTTCGGAGAA-3′. All plasmids were verified by sequencing.

Serial deletion fragments of *SPOP* promoter region (−1320, −1167, −655, −350, −221, −189, −10/+472) were amplified by PCR and cloned into a pGL3-basic luciferase vector (Promega). A *NheI* site was added to the 5′-primer, and a *XhoI* site was added to the 3′-primer. The constructs were verified by sequencing of both DNA strands. The sequences of mutated sites were synthesized by Genescript (Nanjing, China).

FLAG-Gli2, HA-Ub and pcDNA3.1(-)A-RXRA were purchased from GenePharma.

### ChIP-qPCR

ChIP was performed as previously described,^30^ using antibodies for RXRA (Abcam, Cambridge, UK), RNA polymerase II (Abcam), H3Ac (Millipore, Billerica, MA, USA), H3K4 me3 (Millipore), H3K9 me3 (Millipore). The primers were as follows: F: 5′-GCCTCTATTTGGTCCTCGCA-3′, R: 5′-CAAGTCAGGGCCCGGGGATT-3′.

### Luciferase reporter assay

Reporter vectors were transfected into cells together with phRL-TK vectors. After transfection for 48 h, luciferase activity was measured by the Dual Luciferase Assay system (Promega). The phRL-TK vectors were used for standardization of the data. All the procedures were performed in triplicate.

### Ubiquitination assays

Ubiquitination assays of Gli2 were performed by co-transfection of FLAG-Gli2 and HA-Ub. After immunoprecipitation by FLAG antibodies, ubiquitinated Gli2 was detected by HA antibodies in western blot.

### Immunoprecipitation

For immunoprecipitation assays, supernatant of the cell lysates was incubated with indicated antibodies and protein-G agarose beads at 4 °C overnight. The beads were washed with the buffer (0.1% Lubrol-PX, 50 mM KCl, 2 mM CaCl2, 20% glycerol, 50 mM Tris–HCl and inhibitors of proteases and phosphatases, pH 7.4), and the coprecipitated protein was detected with subsequent immunoblotting.

### Cycloheximide protein stability assays

Lovo-NC and Lovo-shSPOP cells were treated with 0.5 mM CHX (Sigma, St. Louis, MO, USA) for different times as indicated, followed by SDS-PAGE and western blotting analysis.

### Electrophoretic mobility shift assays

RXRA protein was obtained when 1 *μ*g of pcDNA3.1(-)A-RXRA or empty vector as DNA template was used according to the protocol of the TNT T7/SP6 Coupled Reticulocyte Lysate System (Promega). EMSA was performed by using LightShift Chemiluminescent EMSA Kit (Pierce Biotechnology, Rockford, IL, USA) following the manufacturer's protocol. The probe sequences are as follows: 5′-CCCGCGCCCTGCGCGCGACCCCCTCCAAGTT-3′. The biotin-labeled oligonucleotide probes were purchased from Sangon (Shanghai, China).

### Anoikis assay

The trypsinized cells (2000 cells) were suspended in 2 ml complete medium plus 0.3% agar (Sigma). The agar–cell mixture was plated as a top layer onto a bottom layer comprising 1% complete medium agar mixture. After 2 weeks of incubation, colonies with size >0.1 mm in diameter were counted.

### Apoptosis analysis

Flow cytometry and Terminal-deoxynucleoitidyl Transferase Mediated Nick End Labeling (TUNEL) were used to analyze cell apoptosis. For flow cytometry, the cells were stained with Alexa Fluor 647 Annexin V and 7-AAD (Becton Dickinson, Franklin Lakes, NJ, USA) according to the manufacturer's instructions. The cells were then tested on a FACScan flow cytometer (Becton Dickinson). Upper (late apoptotic cells) and lower (early apoptotic cells) right quadrants are counted. TUNEL assays were conducted using the TUNEL apoptotic cell detection kit (Roche, Basel, Switzerland), according to the manufacturer's instructions.

### Tumor xenografts and bioluminescence imaging

Male BALB/c nude mice (5 weeks old) were purchased from Vitalriver (Nanjing, China). A total of 24 mice were randomly divided into four groups. Lovo-NC, Lovo-shSPOP, HCT116-Vec and HCT116-SPOP cells were injected subcutaneously into the flanks of the mice (10^6^ cells/100 *μ*l per flank). For bioluminescence imaging, mice were injected with 15 mg/ml luciferin dissolved in PBS at a dose of 10 *μ*l luciferin per gram of body weight. Luciferase activity was visualized using the Xenogen IVIS 2000 small-animal *In Vivo* Imaging System (Xenogen Corp., Alameda, CA, USA). Mouse protocols were approved by the Animal Care and Use Committee of Nantong University.

### Statistical analysis

Each experiment was repeated at least three times throughout the study. Data were reported as the mean±S.D. The correlation between SPOP and Gli2 expression was examined by Spearman correlation test. Statistical analysis was performed with SPSS software (SPSS Standard version 13.0; SPSS, Chicago, IL, USA). *P*-value <0.05 was considered statistically significant.

## Figures and Tables

**Table 1:** Clinical characteristics and SPOP methylation in 118 CRC patients

**Clinical characteristics**	**Number**(***n*****=118)**	**SPOP methylation**		***P***
		**Methylated**(***n*****=31)**	**Unmethylated**(***n*****=87)**	
*Age (years)*				
⩽60	45	13	32	0.669
>60	73	18	55	

*Gender*				
Male	67	14	53	0.144
Female	51	17	34	

*Differentiation*				
Well/moderate	60	13	47	0.298
Poor	58	18	40	

*TNM stage*				
Stage I	26	12	14	0.013*
Stage II/III	92	19	73	

*Lymph node*				
Positive	53	8	45	0.020*
Negative	65	23	42	

* indicates statistically significant difference
